# Novel Targeting of DNA Methyltransferase Activity Inhibits Ewing Sarcoma Cell Proliferation and Enhances Tumor Cell Sensitivity to DNA Damaging Drugs by Activating the DNA Damage Response

**DOI:** 10.3389/fendo.2022.876602

**Published:** 2022-05-31

**Authors:** Camilla Cristalli, Maria Cristina Manara, Sergio Valente, Evelin Pellegrini, Alberto Bavelloni, Alessandra De Feo, William Blalock, Elisabetta Di Bello, David Piñeyro, Angelika Merkel, Manel Esteller, Oscar M. Tirado, Antonello Mai, Katia Scotlandi

**Affiliations:** ^1^ Laboratory of Experimental Oncology, IRCCS Istituto Ortopedico Rizzoli, Bologna, Italy; ^2^ Department of Drug Chemistry and Technologies, Faculty of Pharmacy and Medicine, Sapienza University of Rome, Rome, Italy; ^3^ Istituto di Genetica Molecolare-Luigi Luca Cavalli Sforza, UOS Bologna, Consiglio Nazionale delle Ricerche (IGM-CNR), Bologna, Italy; ^4^ Josep Carreras Leukaemia Research Institute (IJC), Barcelona, Spain; ^5^ Centro de Investigación Biomedica en Red Cancer (CIBERONC), Madrid, Spain; ^6^ Institució Catalana de Recerca i Estudis Avançats (ICREA), Barcelona, Spain; ^7^ Physiological Sciences Department, School of Medicine and Health Sciences, University of Barcelona (UB), Barcelona, Spain; ^8^ Sarcoma Research Group, Oncobell Program, Bellvitge Biomedical Research Institute (IDIBELL), Centro de Investigación Biomedica en Red Cancer (CIBERONC), Barcelona, Spain; ^9^ Istituto Pasteur-Fondazione Cenci Bolognetti, Sapienza University of Rome, Rome, Italy

**Keywords:** ewing sarcoma, epigenetic therapies, DNA methylation, DNMT inhibitors, DNA damage, Drug synergism, doxorubicin, PARP inhibitors

## Abstract

DNA methylation is an important component of the epigenetic machinery that regulates the malignancy of Ewing sarcoma (EWS), the second most common primary bone tumor in children and adolescents. Coordination of DNA methylation and DNA replication is critical for maintaining epigenetic programming and the DNMT1 enzyme has been demonstrated to have an important role in both maintaining the epigenome and controlling cell cycle. Here, we showed that the novel nonnucleoside DNMT inhibitor (DNMTi) MC3343 induces a specific depletion of DNMT1 and affects EWS tumor proliferation through a mechanism that is independent on DNA methylation. Depletion of DNMT1 causes perturbation of the cell cycle, with an accumulation of cells in the G1 phase, and DNA damage, as revealed by the induction of γH2AX foci. These effects elicited activation of p53-dependent signaling and apoptosis in p53wt cells, while in p53 mutated cells, persistent micronuclei and increased DNA instability was observed. Treatment with MC3343 potentiates the efficacy of DNA damaging agents such as doxorubicin and PARP-inhibitors (PARPi). This effect correlates with increased DNA damage and synergistic tumor cytotoxicity, supporting the use of the DNMTi MC3343 as an adjuvant agent in treating EWS.

## Introduction

Ewing sarcoma (EWS), a malignancy of mesenchymal origin with propensity for metastasis, is the second most common primary tumor of bone and soft tissue in children and young people. Although important insights into EWS pathogenesis have emerged in recent years, EWS patient treatment is still confined to dense multidrug chemotherapy, and the prognosis of high-risk patients remains dismal, with a less than 40% of survival rate at 5 years for patients with metastatic disease at diagnosis and for those who relapse after first-line treatment (1–3). Thus, there is a strong demand from patients, families and physicians for additional therapies, which may derive only from further knowledge of the genetic and biological features of the tumor. From a genetic point of view, EWS is characterized by balanced chromosomal translocations in which a member of the FET gene family is fused with an ETS transcription factor, with the most common fusion being EWS–FLI1 (85% of cases) (4). The tumor is a developmental disease, genetically homogeneous (5–7) but epigenetically heterogeneous (8), with an increased level of epigenetic complexity in individuals with metastatic disease (9). The EWS epigenome is increasingly being investigated to identify promising therapeutic targets. EWS-FLI1, the genetic hallmark of EWS and the driving oncogene (10, 11), is a tumor-specific transcriptional factor, responsible for massive epigenetic reprogramming, by inducing *de novo* Ewing-specific enhancers at GGAA microsatellites and by altering the state of gene regulatory elements (12–14). The unique epigenetic signature of EWS could potentially be reverted by the identification of novel agents targeting epigenetic mechanisms. Several promising agents for the treatment of this disease have been reported at the preclinical level, including inhibitors of histone deacetylases (HDACi) (15–17), lysine-specific histone demethylase 1A (LSD1i) (14, 18), DNA methyltransferases (DNMTi) (9, 19–21) and combinations of these agents (22, 23). DNA methylation is essential for crucial biological processes, such as maintaining genome stability, embryonic development and cell differentiation (24, 25). Disruption of DNA methylation patterns represents a common feature of many forms of cancer (26) and was proven to contribute to EWS pathogenesis (9, 27–29). In humans, DNA methylation is catalyzed by members of the DNMT family of enzymes that transfer a methyl group from S-adenosyl-methionine (AdoMet) to DNA cytosine C5 (30, 31). DNMTs are mainly classified in *de novo* methyltransferases, DNMT3s, and maintenance methyltransferase, DNMT1, required respectively to establish and maintain genomic methylation. In addition, DNMT1 contains motifs that allow the enzyme to localize at replication foci during S phase (30), and the absence of DNMT1 was proved to result in replication fork stalling and activation of the DNA damage response (DDR) (32, 33), leading to cell cycle arrest, mitotic catastrophe and through a mechanism independent of DNA methylation. In this study, we evaluate the efficacy of MC3343, a new quinoline-based DNMTi with high activity against cancer cells, including osteosarcoma (34–37). Pharmacologic manipulation of DNMTs, either through the use of conventional FDA-approved nucleoside inhibitor of DNA methylation, 5-aza-2’-deoxycytidine (5AzadC; decitabine), or through non-nucleoside inhibitors was proven to be sufficient to stop tumor growth, reverse the undifferentiated state, and inhibit the invasiveness of cells, thus supporting the therapeutic value of this approach (38). MC3343 is reported to preferentially bind to DNA in CG-rich regions, and to destabilize the DNMT1–AdoM-et DNA complex and two close analogues of MC3343 were shown to degrade DNMTs *via* proteasome degradation (35, 37). Here, we demonstrated that MC3343 specifically inhibits DNMT1 expression and activity in EWS cells, but it does not affect the methylation status of EWS cells. MC3343 slowed cell proliferation, induced DNA damage and cell death, and acted synergistically with other DNA damaging drugs, such as doxorubicin and PARPi, thereby indicating its potential use as an interesting novel adjuvant therapeutic agent against EWS.

## Results

### The Non-Nucleoside DNMTi MC3343 Reduces DNMT1 Protein Levels and Enzyme Activity but Does Not Affect the DNA Methylation Status of EWS Cells

**Figure 1:**
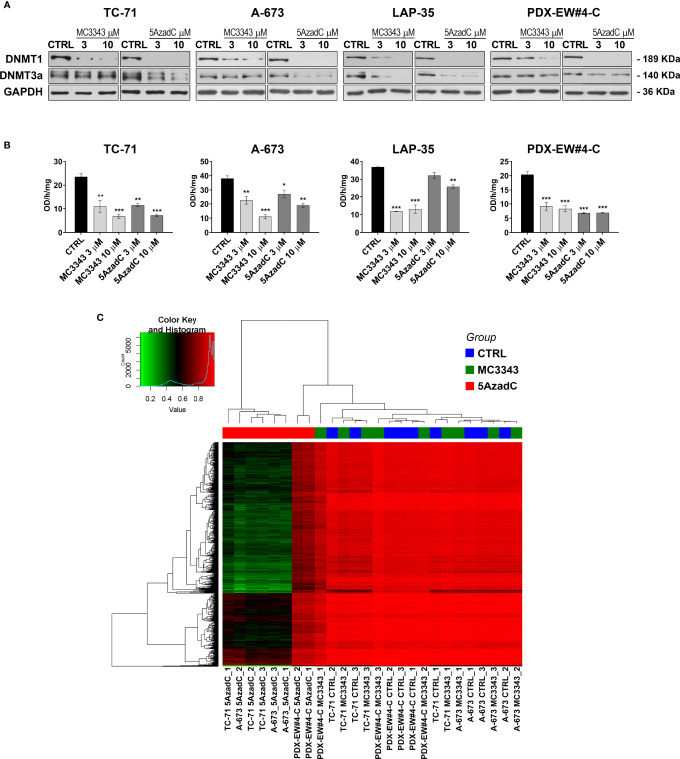
MC3343 efficiently reduces DNMT protein level and enzymatic activity in EWS cells but DNA methylation status is left unaltered. **(A)** Western blot analysis of DNMT1 and DNMT3a, in untreated (CTRL), MC3343-treated (3–10 μM), and 5azadC-treated (3–10 μM) TC-71, A-673, LAP-35 and PDX-EW#4-C cells. Equal protein loading was monitored by anti-GAPDH blotting. One representative immunoblot of three independent experiments is shown. **(B)** DNMT activity measured in EWS cells after MC3343 and 5azadC treatments expressed as OD (optical density)/hours/mg (*p < 0.05; **p < 0.01, ***p < 0.001, one-way ANOVA test). The mean ± SE of three independent experiments are reported. **(C)** Heatmap illustrating the difference of methylation between EWS cells treated with MC3343 and 5azadC.

The efficacy of MC3343 was tested in 11 human patient-derived EWS cell lines, including 8 conventional, and 3 novel cell lines from patient-derived xenografts (PDX). The IC_50_ values, calculated after 72h of cell exposure to the compound, were similar in all the cell lines, ranging from 2 to 5 µM (**Table 1**). The agent induced a dose-dependent reduction of the expression of DNMT1, but the effect on DNMT3a was much milder (**Figure 1A**), indicating a more specific mechanisms of action when compared to the conventional inhibitor of DNA methylation, 5AzadC, that was used as a control. Consistently, MC3343 was similar to or even more potent than 5AzadC in inhibiting DNMT enzyme activity (**Figure 1B**). However, when we used a genome-wide methyl-CpG array to compare the DNA methylation status of three EWS cell lines (TC-71, A-673 and PDX-EW#4-C) after 72h treatment with either MC3343 or 5AzadC, a profound discrepancy between the two drugs was observed. In fact, while cell exposure to 5AzadC resulted in extensive reduction of CpG methylation in all three cell lines, cells treated with MC3343 revealed a methylation pattern indistinct from that observed in untreated cells (**Figure 1C**). This indicates a different mechanism of action between the two compounds that deserves to be further analyzed, because although 5AzadC is approved by the US FDA for the treatment of myelodysplasia and other malignancies, it has demonstrated unsatisfactory results in the treatment of EWS patients (39).

**Table 1:** Cytotoxic effects of the DNMTi MC3343 in EWS cell lines.

Cell line	MC3343 IC_50_ μM
**TC-71**	2.48 ± 0.66
**LAP-35**	5.89 ± 0.06
**6647**	3.99 ± 0.82
**SK-N-MC**	2.96 ± 0.17
**SK-ES-1**	3.96 ± 0.56
**IOR/CAR**	3.83 ± 0.41
**A673**	2.20 ± 0.23
**RD-ES-1**	5.39 ± 0.09
**PDX-EW#2-C**	4.58 ± 0.84
**PDX-EW#4-C**	2.28 ± 0.18
**PDX-EW#5-C**	3.05 ± 0.64

IC_50_ values (drug concentration inducing 50% of growth inhibition) were calculated after 72h exposition to the drug in 11 EWS cell lines. The mean ± SE of three independent experiments are reported.

### DNMTi MC3343 Treatment Results in Reduced Proliferation of EWS Cells and Induction of a Genotoxic Stress Response

**Figure 2:**
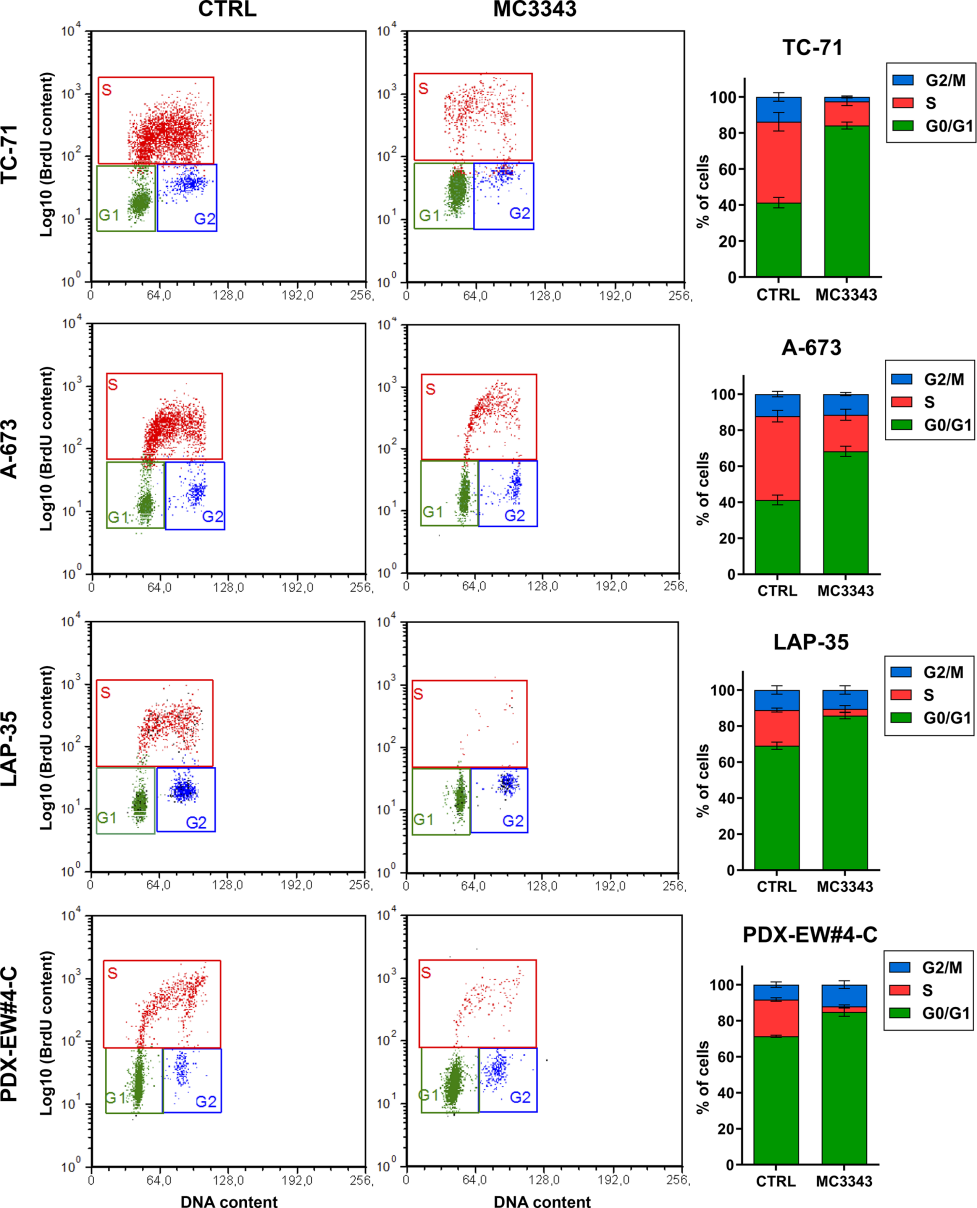
MC3343 induces a G1 block in EWS cells. Cell-cycle analysis of four representative EWS cell lines after 24 h of exposure to MC3343 as determined by BrdU uptake. x-axis indicates DNA content (PI) and y-axis indicates BrdU incorporation (log_10_). In each panel, the different stages of the cell cycle are highlighted. Percentages of cells in each cell-cycle stage are shown in histograms on the right. Data represent the mean ± SE of at least three experiments.

**Figure 3:**
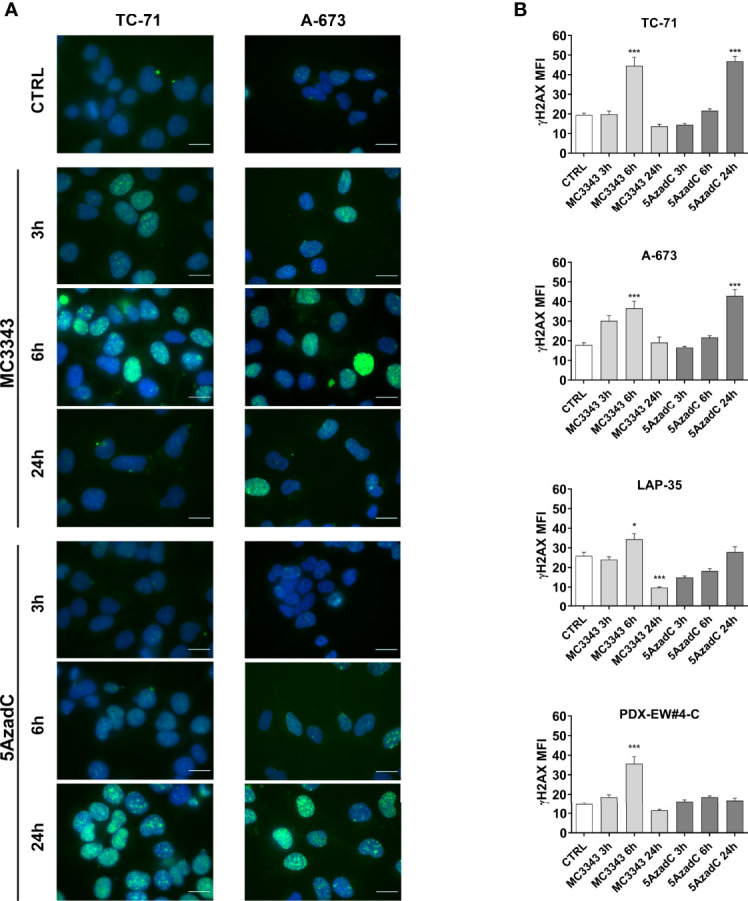
MC3343 and 5azadC kinetics of DNA damage induction. **(A)** Merged immunofluorescence of TC-71 and A-673 staining for γH2AX (green) and DAPI (blue) after 3, 6 and 24h of treatment with DMSO, MC3343 or 5azadC. Digital images were acquired under identical conditions at the same time, using image analysis software (NIS Elements, Nikon). Representative images are shown. Scale bars, 20 µm. **(B)** DNA DSBs measured by levels of mean fluorescence intensity (MFI) of γH2AX positive nuclei in at least 20 different fields for each condition. (*p < 0.05; ***p < 0.001, one-way ANOVA test).

**Figure 4:**
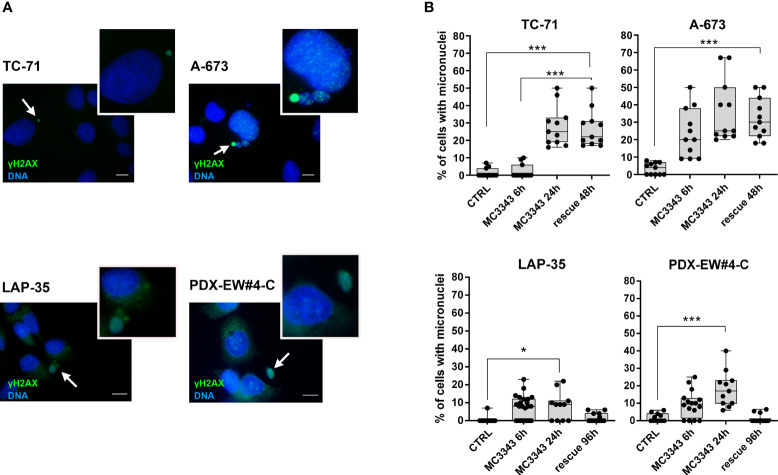
MC3343 treatment induces micronuclei formation. **(A)** Representative images of γH2AX (green) and DNA (Hoechst 33258, blue) staining of EWS cells following treatment with MC3343. The white arrows indicate micronuclei that contains DSBs (as indicated by DNA and γH2AX double-staining). Scale bars, 10 µm. **(B)** Percentage of micronuclei in EWS cells treated with MC3343 at the indicated times and after 48-96h of rescue with drug-free medium replacement. Data are presented as the mean percentage of micro-nucleated cells quantified from at least 20 random fields (mean ± SE; *p < 0.05, ***p < 0.001, one-way ANOVA test).

**Figure 5:**
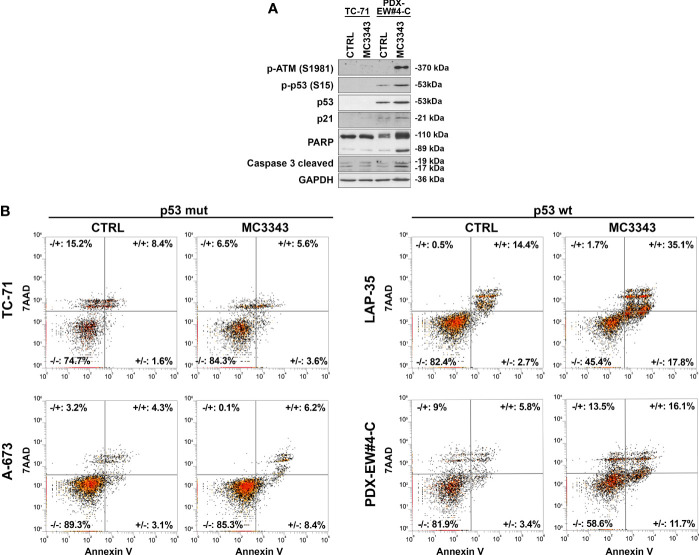
Activation of p53 signaling and induction of apoptosis in *TP53* wild type cell lines. **(A)** *TP53* mutated TC-71 cell line and *TP53* wild-type PDX-EW#4-C cells were treated with 3µM MC3343 for 24h. Whole cell lysates were analyzed using western blot. Three independent experiments were performed and one representative immunoblot of p-ATM, p21, phospho-p53, p53, PARP and caspase-3 cleaved is shown **(B)** Representative flow cytometry scatter plots showing apoptosis of p53 mutated (TC-71 and A-673) and p53 wild-type (LAP-35 and PDX-EW#4-C) EWS cells stained with Annexin-V/7-AAD following 48h treatment with MC3343 (3-5 µM) or DMSO. Numbers at the corners represent the percentage of cells found in each quadrant (viable-lower left, early apoptotic-lower right, late apoptotic-upper right, and dead cells-upper left are indicated).

The depletion of DNMT1 leads to the removal of the enzyme from replication forks, which was demonstrated to result in DNA strand breaks and the activation of genotoxic stress pathway signaling, leading to further DNA damage, γH2AX foci formation and cell death (40, 41). Thus, we analyzed the impact of MC3343 treatment on EWS cells on these biological processes. Using double labeling with propidium iodide to mark S-phase cells and bromodeoxyuridine (BrdU) to label cells actively synthesizing DNA, we discovered that MC3343 treatment triggers a significant accumulation of cells in the G1 (**Figure 2**). As expected, the percentage of cells in G1 phase was higher in the faster proliferating TC-71 and A-673 cells (doubling time: 18-20h) compared to LAP-35 and PDX-EW#4-C (doubling time: 40-50h) (p < 0.05 Student’s t test). Besides effects on the cell cycle, MC3343 induced the formation of histone variant γH2AX foci, a characteristic marker of cells undergoing replication stress and DNA strand breaks (42) with much faster kinetics as compared to 5AzadC. In fact, treatment of EWS cells with MC3343 led to an induction of γH2AX foci starting after 3h with a peak at 6h; while in cells treated with 5AzadC, the appearance of γH2AX foci formation occurred only after 24h and not in all cell lines (**Figure 3** and **Supplementary Figure S1**). We also evaluated the impact of MC3343 on genomic instability in four EWS cell lines. Control and treated cells were cultured for 6 to 24 hours, and the impact on genome stability monitored by evaluating micronuclei, a well-recognized marker of genomic instability (43) (**Figures 4A, B**). This effect was better represented in p53 mutated TC-71 and A-673 cells than in p53wt LA-P35 and PDX-EW#4-C cells (p< 0.0001 Student’s t test). In addition, the percentage of cells with micronuclei following exposure to MC3343 was maintained in p53 mutated cells even after removing the compound for at least two cell doublings (rescue for 48h-96h), indicating that in p53 mutated cells (TC-71 and A-673) micronuclei are inherited and persist into the daughter cells. In contrast, when the p53 response is functional (LAP-35 and PDX-EW#4-C), exposure to MC3343 led to activation of p53-dependent signaling (**Figure 5A**) and cells that were more prone to apoptosis, as demonstrated by annexin labeling (**Figure 5B**). Representative western blottings indicated that inactivation of DNMT1 by MC3343 induced phosphorylation of p53 on Ser15, a residue that is targeted by ATM and whose phosphorylation has been correlated with accumulation of p53 and activation of its downstream targets in response to DNA damage (44). Moreover, upregulation of p21 and cleavage of pro-Caspase 3 and PARP1 were consistently observed in p53 wt MC3343-treated PDX-EW#4-C but not in p53 mut TC-71 cells.

### The DNMT1 MC3343 Inhibitor Synergistically Increases the Cytotoxicity of Doxorubicin and PARPi in EWS Cells

**Figure 6:**
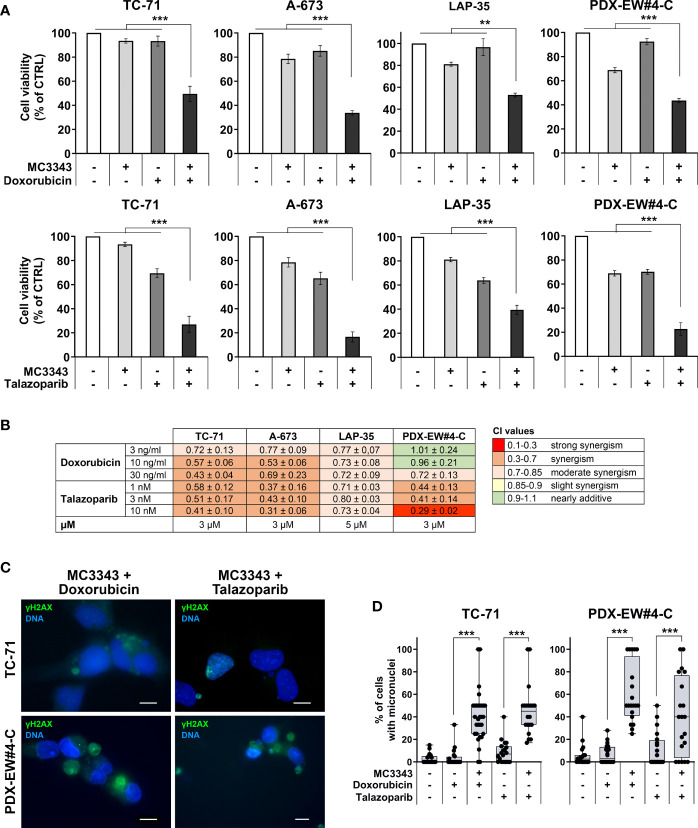
Synergism of combined treatments with DNA damaging drugs. **(A)** TC-71, A-673, LAP-35 and PDX-EW#4-C cells were treated for 24h with 3-5 µM MC3343 and, after wash-out, treated for 48h with 3-10 ng/mL of doxorubicin or 3-10 nM of talazoparib. Cell viability was measured by trypan blue vital cell counts and is expressed as the percentage of live cells with respect to the untreated control. Data are shown as mean ± SE of three independent experiments (*p < 0.05; **p < 0.01, ***p < 0.001, one-way ANOVA test). **(B)** Heatmap of drug interactions of sequential treatments with MC3343 and doxorubicin or talazoparib. Cell viability was assessed by trypan blue cell counts. Synergistic, additive, or antagonistic drug interactions were calculated by combination index (CI). **(C)** Merged immunofluorescence of EWS cells staining for γH2AX (green) and DNA (blue) after treatment with MC3343 and doxorubicin/talazoparib as indicated. Representative images are shown. Scale bars, 10 µm. **(D)** Percentage of micro-nucleated cells after sequential treatment with MC3343 and doxorubicin or talazoparib. Data are presented as mean percentage of micro-nucleated cells quantified from at least 20 random fields (mean ± SE; ***p < 0.001, one-way ANOVA test).

We then evaluated the impact of the DNMTi MC3343 on cytotoxicity induced by DNA damaging agents, such as doxorubicin, a widely-used genotoxic drug that intercalates between DNA base pairs and inhibits the enzyme topoisomerase II (45), and talazoparib, a PARPi that functions by impairing DNA repair mechanisms (46, 47). The pretreatment of cells with the MC3343 increased the cytotoxicity of both doxorubicin and talazoparib (**Figure 6A**). Combination indexes ranged from moderate to strong synergism (**Figure 6B**) and the formation of micronuclei that are prone to rupture (γH2AX positive) was significantly higher in combined treatments (**Figures 6C, D**).

## Discussion

EWS is a developmental tumor that derives from the transformation of an undifferentiated mesenchymal stem cell that is blocked during the differentiation process by the occurrence of a reciprocal chromosomal translocation and forming of an EWS-ETS chimera. The quiet nature of the EWS genome, the paucity of mutations, and the evidence that EWS-FLI1 can induce a complete reprogramming of gene expression (12, 13) guided the attention of researchers to epigenetic alterations as the main mechanisms capable of regulating progression of this disease. Among the different epigenetic mechanisms, DNA methylation was demonstrated to have an important role leading to patterns of enhancer reprogramming that were shared by all EWS samples (9). The importance of DNA methylation alterations in EWS tumorigenesis and progression together with exciting outcomes in hematological malignancies (21, 48, 49), encouraged us to study the therapeutic potential of DNMT inhibitors in EWS. Unlike genetic alterations, DNA methylation is reversible, which makes it extremely interesting for therapeutic approaches. However, although several epi-drugs received FDA approval (39), epigenetic therapy is still in its early stages. 5-azacytidine (5-AZA) and 5AzadC, first synthesized by Piskala and Sorm (1964), are known to be incorporated into DNA and to inhibit DNA methylation, which has led to the use of Decitabine as a first line therapeutic agent for cancers in which epigenetic silencing of critical regulatory genes has occurred (50). Nevertheless, despite their high efficacy, such drugs have demonstrated poor bioavailability, chemical instability, and toxic side-effects in phase I trials, supporting the need for development of other, more effective and less toxic compounds. The quinoline derivative MC3343 specifically targets DNMT1, and we have recently demonstrated that this novel non-nucleoside DNMTi affects osteosarcoma cell proliferation, induces osteoblastic differentiation through specific re-expression of genes regulating this physiologic process and increased stable doxorubicin binding to DNA with fewer toxic effects on healthy cells than either 5-AZA or 5azadC (34). Here, we demonstrated that although MC3343 reduced DNMT1 protein levels and enzymatic activity in all EWS cell lines tested, the compound had little effects on the DNA methylation status of treated cells. This effect was remarkably different with respect to 5AzadC as but similar to what was previously reported in studies in where DNMT1 knock-down was obtained by either antisense oligonucleotides or siRNA approaches (51–53). The genetic inactivation of DNMT1 was found to activate a number of genes involved in the cellular stress response and cell cycle arrest by a DNA methylation-independent mechanism (54). In EWS cells, disruption of DNMT1 induced by MC3343 resulted in the loss of cell proliferation, followed by progressive DNA instability and cell death. Upon inactivation of DNMT1, cells arrest in the G1 phase, leading to blockage of DNA replication. DNMT1 is normally recruited to the replication fork, and previous data suggested that it is the absence of the DNMT1 protein from the fork, not the loss of its DNA methylation activity, that causes stalled forks and activation of the DNA damage response (52). Accordingly, we observed DNA double strand break (DSB) formation (γH2AX foci) after treatment with MC3343. DNA damage led to the accumulation of cells with persistent DNA instability in p53 mutated cells, as shown by increased micronuclei, while in wt p53 cells, which are most representative of the condition in EWS (7), MC3343 led to the activation of p53 and induction of apoptosis. In addition, pharmacologic targeting of DNMT1 by MC3343 was found to potentiate the efficacy of cytotoxic agents, such as the conventional chemotherapeutic doxorubicin and the PARPi talazoparib. Doxorubicin is a leading drug in the treatment of EWS but fails as a single agent; while PARPi were found to be highly effective in preclinical conditions (55), but they have failed to show impressive clinical benefits for patients with EWS (56), suggesting the need to develop new strategies to maximize the effectiveness of these agents. We previously demonstrated that MC3343, as well as siRNA against DNMTs, was capable of increasing stable doxorubicin bonds with DNA, triggering doxorubicin-induced DNA damage (34). Others have shown that pretreatment with DNMTi also leads to enhanced tight binding of PARP1 to chromatin, enhancing DNA damage and PARPi efficacy (57). In this paper we confirmed that the combination of the DNMTi MC3343 and DNA damaging agents potentiates effects against EWS.

Taken together, we demonstrate in the present study that DNMT1 is essential for the proliferation and survival of EWS cells. Complete inactivation of DNMT1 by MC3343 results in a G1 phase arrest and triggers the activation of DDR. Very likely, p53 mutated cells that escape cell cycle arrest enter mitosis with their damaged DNA largely unrepaired, which causes severe abnormalities, culminating in cell death. In p53 wt cells, p53 activation following replication disruption led to apoptosis. In keeping with previous evidences (51–53), this response appears to be independent of the effects of DNMT1 in DNA methylation. The fact that different methods of DNMT1 knockdown targeting different regions of DNMT1 as well as pharmacological inhibition trigger the same response in a broad panel of cells from both human and mouse supports the hypothesis that depletion of DNMT1 is mainly responsible for the general cellular response reported here. The demonstration that MC3343 treatment increases the efficacy of doxorubicin and PARPi in killing EWS cells offers the possibility of reducing the toxicity of these agents while improving therapy, which represents a valuable extra benefit for pediatric patients with EWS.

## Materials and Methods

### Chemicals

MC3343 was prepared as previously reported (36, 37). Stock solutions of 5-Aza-2′-deoxycytidine (5azadC) (A3656, Sigma-Aldrich, St. Louis, MA, U.S.), Doxorubicin (D1515, Sigma-Aldrich) and Talazoparib (BMN673) (S7048, Selleckchem, Houston, TX, U.S.) were prepared and stored according to the manufacturer’s instructions.

### DNMT Activity Assay in a Cell Context

DNMT activity was quantified with an EpiQuick DNA Methyltransferase Activity/Inhibition Assay Kit (P-3001-1, Epigentek Inc., Farmingdale, NY, U.S.). Cells were treated with MC3343 or 5azadC (3-10 µM) for 48 h, and 5µg of nuclear extracts, isolated with EpiQuick Nuclear Extraction Kit (OP-0002-1, Epigentek Inc), were added to each reaction well, according to the manufacturer’s protocol. Absorbance was determined using a microplate spectrophotometer at 450 nm (GloMax luminometer, Promega, Madison, WI, U.S.). The data are expressed as the mean ± SE of three independent experiments.

### Cells Lines

A panel of 11 EWS human cell lines were analyzed. A-673 (CRL-1598), SK-ES-1 (HTB-86), SK-N-MC (HTB-10), and RD-ES (HTB-166) were provided by the American Type Culture Collection (ATCC, Manassas, VA, U.S.); TC-71 and 6647 were kindly provided by T.J. Triche (Children’s Hospital, Los Angeles, CA, U.S.). LAP-35 and IOR/CAR, were obtained in the Experimental Oncology Lab of the Rizzoli Institute as previously described (58, 59). The cell lines PDX-EW#2-C, PDX-EW#4-C and PDX-EW#5-C were obtained from patient-derived xenografts (PDXs) as previously described (60). All cell lines were tested for mycoplasma contamination every 3 months (LT07-318, MycoAlert Mycoplasma Detection Kit, Lonza, Basel, Switzerland). Cell lines were authenticated (STR profiling) by analysis of the following loci: AMEL, D3S1358, TH01, D21S11, D18S51, D10S1248, D1S1656, D2S1338, D16S539, D22S1045, VWA, D8S1179, FGA, D2S441, D12S391, D19S433 and SE33 (last control December 2017 and July 2018; POWERPLEX ESX 17 Fast System, Promega, Madison, WI, U.S.). All cell lines were immediately amplified to constitute liquid nitrogen stocks and were never passaged for more than 1 month upon thawing. Cells were maintained in Iscove’s Modified Dulbecco’s Medium (ECB2072L, IMDM, Euroclone, Milan, Italy) supplemented with 10% heat-inactivated FBS (ECS0180L, Euroclone), penicillin (20 U/mL) and streptomycin (100 µg/mL) (ECB3001D, Euroclone) in a 37°C humidified at 5% CO_2_.

### Cell Treatments

To perform cell viability experiments, 2-5x10^5^ cells were plated in 6-well dishes and treated after 24h with the indicated drugs for 72h, roughly equivalent to at least two doubling times of each cell line, before Trypan blue (T8154, Sigma-Aldrich) vital cell counting or DNA methylation studies. In parallel, cells were treated with medium containing dimethyl sulfoxide (DMSO) (D2438, Sigma-Aldrich) as a control. The highest final concentration of DMSO in the medium was <0.005%, which had no effect on cell growth.

In sequential drugs combination experiments, cells were plated and pre-treated with MC3343 (3-5 µM) for 24h then the medium was replaced with the drugs at indicated concentrations (doxorubicin 3-30 ng/ml, talazoparib 1-10 nM) or fresh IMDM medium without drugs.

### Flow Cytometry

For cell cycle analysis after 24h of treatment, 5-bromo-2’-deoxyuridine (BrdU) (B5002, Sigma-Aldrich) labeling was used to assess cell proliferation. Cells were processed for indirect immunofluorescence using anti‐BrdU mAb (1:8, 347580, BD-Biosciences, Franklin Lakes, NJ, U.S.) primary antibody and anti‐mouse FITC secondary antibody (1:100, 31569, Thermo Fisher Scientific, Waltham, MA, U.S.) and 20 μg/mL propidium iodide (P4170, Sigma-Aldrich) for the analysis of DNA content prior to flow cytometry analysis (Sysmex, Partec GmbH Münster, Germany). Detection and quantification of apoptosis was obtained by cell staining with Annexin V and 7-amino-actinomycin D (7-AAD) (A1310, Thermo Fisher Scientific) using the PE Annexin V detection kit I (Life Technologies Corporation, Monza, Italy) according to the manufacturer’s instructions. Samples were analyzed on an Attune NxT Acoustic Focusing Cytometer (Life Technologies Corporation), and data were analyzed using Attune Cytometric 2.6 software (Life Technologies Corporation). Cells were classified as early apoptotic, late apoptotic or dead if they were Annexin V+/7-AAD−, Annexin V+/7-AAD+ or Annexin V−/7-AAD+, respectively.

### DNA Extraction and Methylation Profiling

DNA was extracted from cell pellets by PureLink genomic DNA kit (K182001, Thermo Fisher Scientific). The DNA methylation profiles of the samples were obtained using the Infinium Methylation EPIC Array (850 K). DNA samples were assessed for their quality using a NanoDrop^®^ ND-2000 UV-Vis Spectrophotometer (Thermo Fisher Scientific). The samples were separated in agarose gels. Those with intact genomic DNA, showing no smear in the gel, were selected for subsequent experiments. Intact genomic DNA was diluted to 50 ng/μL based on Quant-iT PicoGreen (Invitrogen) quantitation. Concentrations were adjusted according to these results. For bisulfite conversion, 600 ng of input gDNA was required. Bisulfite-modified gDNA was prepared using the EZ DNA Methylation kit (Zymo Research) according to the manufacturer’s instructions. Conversion reagent was added, followed by subsequent incubation in a thermocycler to denature the samples. CT-converted DNA was washed and de-sulfonated with de-sulfonation buffer, after which the DNA was washed again and eluted with 12 μL elution buffer. The whole-genome amplification process required 250 ng of input bisulfite-converted DNA (MA1) and created a sufficient quantity of DNA (1,000X amplification) for use on a single BeadChip in the Infinium methylation assay (Illumina RPM and MSM). After amplification, the product was fragmented using a proprietary reagent (FMS), precipitated with 2-propanol (plus precipitating reagent; PM1), and re-suspended in formamide-containing hybridization buffer (RA1). The DNA samples were denatured for 20 min at 95°C and placed in a humidified container for a minimum of 16 h at 48°C, allowing CpG loci to hybridize with the 50-mer capture probes. Following hybridization, the BeadChip/Te-Flow chamber assembly was placed on a temperature-controlled Tecan flow-through chamber rack, and subsequent washing, extension, and staining were performed by adding reagents to the Te-Flow chamber. For the allele-specific single-base extension assay, primers were extended by polymerase and labeled nucleotide mix (TEM), and then stained by repeated application of STM (staining reagent) and ATM (anti-staining reagent). After staining, the slides were washed with low-salt wash buffer (PB1), immediately coated with XC4, and imaged using the iScan System (Illumina). The iScan System has a two-color (532 nm/658 nm) confocal fluorescent scanner with 0.54 μm pixel resolution. The scanner excited the fluorophores generated during signal amplification/staining of the allele-specific (one color) extension products on the BeadChips. Image intensities were extracted using Illumina’s GenomeStudio Software.

### DNA Methylation Analysis

Methylation array data was processed with R statistical language using methods from the Bioconductor. Data quality was assessed using the standard pipeline from the minfi package: Low quality probes (detection pvalue > 0.01) and low-quality samples (> 0.05% low quality probes) were removed from the analysis and known SNPs, sex chromosomes and cross-hybridizing probes were excluded. The data was normalized using ‘ssNoob’ normalization method and, after normalization, m-values and beta-values were calculated. Differential methylation analysis was performed with the limma package (~ 0 + treatment + cell line; single sites, p<0.01) and DMRcate (regions, fdr < 0.01).

### Immunofluorescence Analysis

Immunofluorescence was performed on adherent cells grown on coverslips, fixed in cold methanol for 7 minutes at -20°C and permeabilized with 0.15% Triton X-100 (T8787, Sigma-Aldrich) in PBS, blocked with 4% Bovine serum albumin (BSA) (A4503, Sigma-Aldrich)/PBS for 1 hour at room temperature and incubated with anti-phospho-histone H2A.X (Ser139) antibody (1:100, 9718, Cell Signaling Technology, Danvers, MA, U.S). Nuclei were counterstained by bisbenzimide Hoechst 33258 (0.5 µg/ml, B2883, Sigma-Aldrich, St. Louis, MA, U.S.). Cell fluorescence was then evaluated using a Nikon Eclipse 90i microscope (Nikon Instruments, Florence, Italy). For quantification purposes, γH2AX spots and micronuclei were quantified from at least 20 random fields (~500 cells), expressed as γH2AX mean fluorescence intensity (MFI) and percentage of cells with micronuclei respectively. Images acquisition and processing were conducted using the NIS-Elements A.R. 3,10 software.

### Western Blotting

EWS cells were lysed with RIPA buffer (89900, Thermo Fisher Scientific) containing protease inhibitors (A32955, Thermo Fisher Scientific) and phosphatase inhibitors (A32957, Thermo Fisher Scientific). Equivalent amounts of total cell lysates were separated by 8-12% SDS-PAGE under denaturing conditions and transferred onto nitrocellulose membrane (0.45 μm, Bio-Rad, Hercules, CA, U.S.). Membranes were incubated overnight with the following primary antibodies: anti-DNMT1 (1:2000, A300-041A-M lot# 1, Bethyl Laboratories, Montgomery, TX, U.S), anti-DNMT3a (1:1000, sc-20703 lot # L0412, Santa Cruz Biotechnology, Dallas, TX, U.S.), anti-phospho-ATM (S1981) (1:2000, ab36810 lot# gr3185919-27, Abcam, Cambridge, UK), anti-p21 (1:1000, sc-6246 lot # E0511, Santa Cruz Biotechnology), anti-phospho-p53 (Ser15) (1:2000, 9284 lot#9, Cell Signaling Technology), anti-p53 (1:3000, MCA1701 lot #410, Bio-Rad), anti-PARP1 (1:5000, 9542 lot#15, Cell Signaling Technology), anti-cleaved Caspase-3 (Asp175) (1:500, 9661 lot # 38, Cell Signaling Technology) and anti-GAPDH (1:10000, 2118 lot # 14, Cell Signaling Technology). After washes with 1x Tris-buffered saline/Tween20, the membranes were incubated with secondary anti-rabbit or anti-mouse antibodies conjugated to horseradish peroxidase (GE Healthcare, Piscataway, NJ, U.S.) and revealed by SuperSignal West Pico PLUS Chemiluminescent Substrate (34580, Thermo Fisher Scientific).

### Statistical Analyses

All statistical analyses were performed using Prism version 7.0 (GraphPad Software, La Jolla, CA, US). IC_50_ (concentration required to inhibit cell proliferation by 50%) values were calculated from the linear transformation of dose-response curves. Comparison between two groups were evaluated with two-tailed Student’s t-tests. Experimental data including more than 2 groups were analyzed using one-way or two-way ANOVA. The data were considered statistically significant at p <0.05. Drug interactions were analyzed by the combination index (CI) calculated with the fractional product method based on that described by Chou et al. (61). Subclassification of CI values was used (0.1– 0.3 strong synergism, 0.3–0.7 synergism, 0.7–0.85 moderate synergism, 0.85–0.9 slight synergism, 0.9–1.1 nearly additive).

## Data Availability Statement

The data presented in the study have been deposited in NCBI's Gene Expression Omnibus accession number GSE202143 (https://www.ncbi.nlm.nih.gov/geo/query/acc.cgi?acc=GSE202143).

## Ethics Statement

The ethics committee of the Rizzoli Institute approved the study (Prot. 0006167 2020/04/29; Prot. 0011371 2019/09/25) and the establishment of PDX models (Prot. 0009323 2016/04/22 and Prot. 0009164 2017/09/22).

## Author Contributions

Conception and design: CC and KS. Development of methodology: CC, MM, SV, DP, ME, AMe, OT, AMa, KS. Acquisition and analysis of data: CC, MM, EP, AB, AD, WB, ED, DP, AMe, ME, OT. Writing, review, and/or revision of the manuscript: KS, CC, and MM wrote the manuscript, and all authors reviewed and edited the manuscript. Study supervision: CC and KS. All authors contributed to the article and approved the submitted version.

## Funding

This work was supported by the Italian Ministry of Health (RF-2016-02361373).

## Conflict of Interest

The authors declare that the research was conducted in the absence of any commercial or financial relationships that could be construed as a potential conflict of interest.

## Publisher’s Note

All claims expressed in this article are solely those of the authors and do not necessarily represent those of their affiliated organizations, or those of the publisher, the editors and the reviewers. Any product that may be evaluated in this article, or claim that may be made by its manufacturer, is not guaranteed or endorsed by the publisher.
