# Supplementary figures and images for: Novel Targeting of DNA Methyltransferase Activity Inhibits Ewing Sarcoma Cell Proliferation and Enhances Tumor Cell Sensitivity to DNA Damaging Drugs by Activating the DNA Damage Response
Source: Front Endocrinol (Lausanne). 2022 May 31;13:876602. doi: 10.3389/fendo.2022.876602 (PMC9197596; doi:10.3389/fendo.2022.876602)

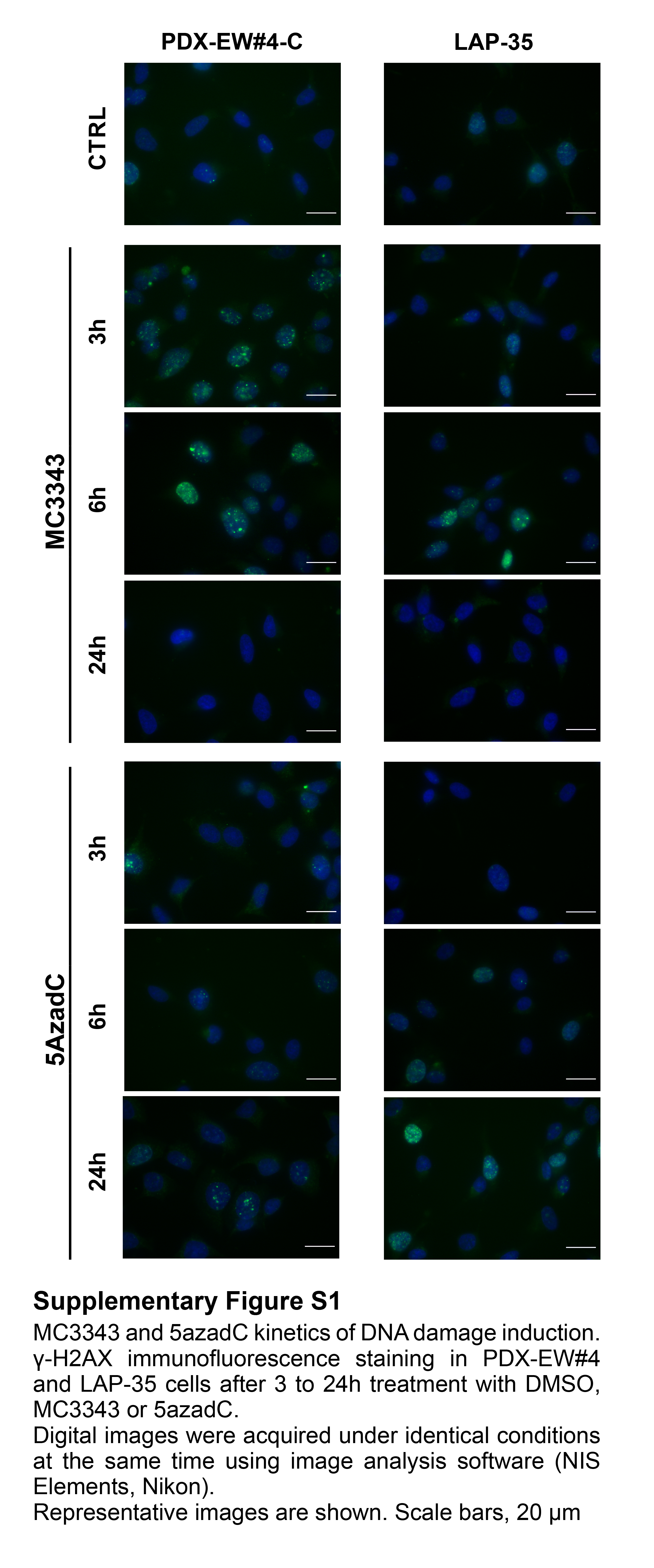

Supplement: Supplementary file 1 [file Image_1.tif]
